# Quantitative Bone SPECT/CT of Central Cartilaginous Bone Tumors: Relationship between SUVmax and Radiodensity in Hounsfield Unit

**DOI:** 10.3390/cancers16111968

**Published:** 2024-05-22

**Authors:** Hyukjin Yoon, Seul Ki Lee, Jee-Young Kim, Min Wook Joo

**Affiliations:** 1 Division of Nuclear Medicine, Department of Radiology, St. Vincent’s Hospital, College of Medicine, The Catholic University of Korea, Seoul 06591, Republic of Korea; 2 Department of Radiology, St. Vincent’s Hospital, College of Medicine, The Catholic University of Korea, Seoul 06591, Republic of Korea; 3 Department of Orthopaedic Surgery, St. Vincent’s Hospital, College of Medicine, The Catholic University of Korea, Seoul 06591, Republic of Korea

**Keywords:** cartilaginous bone tumor, bone SPECT/CT, SUVmax, hounsfield units, chondroid matrix mineralization, correlation

## Abstract

**Simple Summary:**

Radionuclide bone imaging, which reflects osteoblastic activity, is used in evaluating cartilaginous bone tumors; higher SUVmax is more indicative of an ACT rather than an enchondroma in SPECT/CT. However, SUVmax can be influenced by several factors, including radiodensity. Therefore, this study was designed to correlate radiodensity measurements with SUVmax of central cartilaginous bone tumors, including enchondroma, and low-to-intermediate grade chondrosarcomas. Our findings revealed a significant negative correlation between SUVmax and radiodensity measurements in HUmax, HUmean, and HU_SD_. The subgroup analysis showed significantly higher SUVmax and lower HU_SD_ in the malignant group (grade 1 and 2 chondrosarcoma) than in the benign group (enchondroma). It was observed that higher SUVmax and lower HU_SD_ were associated with a higher probability of having a low-to-intermediate grade chondrosarcoma with aggressive features and a less calcified tumor matrix.

**Abstract:**

(1) Background: it is challenging to determine the accurate grades of cartilaginous bone tumors. Using bone single photon emission computed tomography (SPECT)/computed tomography (CT), maximum standardized uptake value (SUVmax) was found to be significantly associated with different grades of cartilaginous bone tumor. The inquiry focused on the effect of the tumor matrix on SUVmax. (2) Methods: a total of 65 patients from 2017 to 2022 with central cartilaginous bone tumors, including enchondromas and low-to-intermediate grade chondrosarcomas, who had undergone bone SPECT/CT were retrospectively enrolled. The SUVmax was recorded and any aggressive CT findings of cartilaginous bone tumor and Hounsfield units (HU) of the chondroid matrix as mean, minimum, maximum, and standard deviation (SD) were reviewed on CT scans. Pearson’s correlation analysis was performed to determine the relationship between CT features and SUVmax. Subgroup analysis was also performed between the benign group (enchondroma) and the malignant group (grade 1 and 2 chondrosarcoma) for comparison of HU values and SUVmax. (3) Results: a significant negative correlation between SUVmax and HU measurements, including HUmax, HUmean, and HU_SD_, was found. The subgroup analysis showed significantly higher SUVmax in the malignant group, with more frequent CT aggressive features, and significantly lower HU_SD_ in the malignant group than in the benign group. (4) Conclusions: it was observed that higher SUVmax and lower HU_SD_ were associated with a higher probability of having a low-to-intermediate chondrosarcoma with aggressive features and a less calcified tumor matrix.

## 1. Introduction

Cartilaginous bone tumors are among the most common bone tumors [1]. Enchondroma represents the most prevalent benign tumor and chondrosarcoma represents the most common malignant tumor [2]. Distinguishing between the grades of these tumors, particularly between enchondroma and atypical cartilaginous tumor (ACT), is often challenging due to their similar radiological and histologic features [2,3,4,5,6]. While most enchondromas do not necessitate treatment unless symptomatic or causing complications, ACTs require curettage due to their locally aggressive nature [7]. Thus, efforts have been made to improve radiological differentiation between these tumors using imaging modalities like computed tomography (CT) or magnetic resonance imaging (MRI) [8].

Radionuclide bone imaging, which reflects osteoblastic activity, is also used in evaluating cartilaginous bone tumors. Higher-grade tumors typically exhibit an increased uptake on scintigraphy, reflecting cortical destruction and permeation due to cartilaginous tumor growth [9]. Single photon emission computed tomography (SPECT) provides three-dimensional information on radiotracer uptake, while the combination of SPECT and CT (SPECT/CT) enables accurate localization of the uptake [10]. Recent advancements have allowed for quantitative analysis of radiotracer distribution, with maximum standardized uptake value (SUVmax) being widely used in clinical practice due to its simplicity [10,11]. A previous study by Choi et al. suggests that a higher SUVmax is more indicative of an ACT rather than an enchondroma, with a cutoff value of 15.6 [10].

However, SUVmax can also be high in enchondromas, raising questions about other factors influencing it besides cortical destruction and permeation. While previous studies have focused on imaging features determining tumor grading, such as endosteal scalloping, cortical expansion, and disruption [8,12,13,14], the significance of chondroid matrix mineralization within the tumor has not been thoroughly explored. Cartilaginous bone tumors often exhibit chondroid matrix mineralization [2], which can be analyzed by measuring the radiodensity using the Hounsfield units (HU) scale on CT, indicating strength and distribution [15]. Given a report suggesting that bone mineral density (BMD) influences bone SPECT/CT radiotracer uptake [16], we planned to investigate the relationship between radiodensity on CT and SUVmax of a central cartilaginous bone tumor on SPECT. 

Therefore, our aim is to investigate the relationship between chondroid matrix mineralization and bone radionuclide uptake in central cartilaginous bone tumors. Combined SPECT/CT images provide both radiodensity of HU information from CT and SUVmax from SPECT simultaneously, making it an excellent tool for analysis. Also, we performed subgroup analysis for comparison of radiodensity in HU measurements and SUVmax between benign and malignant groups. 

## 2. Materials and Methods

### 2.1. Patient Selection

This retrospective study was approved by the institutional review board at our institution and informed consent was waived. From July 2017 to December 2022, 108 patients with suspected cartilaginous bone tumors underwent bone SPECT/CT. Moreover, 37 patients with suspected cartilaginous bone tumors in the hand or foot were excluded from the analysis in order to avoid a selection bias due to the different radiological and histopathologic appearances that may falsely suggest aggressiveness (such as pathologic fracture), particularly in small bones, even if the tumors were benign and a disproportionate amount of enchondromas was present in these regions. Five patients with secondary chondrosarcomas arising from osteochondroma or enchondromatosis and one patient with dedifferentiated chondrosarcoma were also excluded. Accordingly, 65 patients (22 males, 43 females; mean age 52.7 ± 14.7 years; range 18–83 years) with the diagnosis of an enchondroma, ACT/chondrosarcoma grade 1 (CS1, low grade), and chondrosarcoma grade 2 (CS2, intermediate grade) were included in the analysis. All diagnoses were made by pathological findings (n = 27) via surgery or biopsy with preoperative imaging studies and by clinical and radiological findings (n = 38) such as X-ray, CT, or MRI without pathological confirmation due to a high suspicion of benign conditions. In none of these patients was the diagnosis changed during follow-ups of at least two years. Clinical and radiological information such as age, gender, and tumor location were obtained from medical records. Patients had central cartilaginous bone tumors in the proximal humerus (n = 20; 18 enchondromas and 2 ACTs), distal femur (n = 28; 19 enchondromas, 8 ACTs, and 1 CS2), proximal femur (n = 7; 4 enchondromas, 2 ACTs, and 1 CS2), proximal fibula (n = 4; 2 enchondromas and 2 ACTs), distal radius (n = 1, ACT), scapula (n = 2; 1 enchondroma and 1 CS2), and pelvic bone (n = 3; 1 enchondroma, 1 CS1, and 1 CS2). 

### 2.2. Bone SPECT/CT Acquisition

All bone SPECT/CT scans were conducted using an NMCT/670 SPECT/CT scanner (GE Healthcare, Waukesha, WI, USA). First, 800–1100 MBq of Tc-99m hydroxymethylene diphosphonate (HDP) was injected. SPECT/CT images of the tumor site were obtained 4 h after the radiotracer injection. CT acquisition was done with the following parameters: peak energy at 140 keV with 10% window and step-and-shot mode acquisition (25 s per step and 30 steps per detector) with 6° angular increments. For SPECT image reconstruction, an iterative ordered subset expectation maximization algorithm was employed (four iterations; 10 subsets), with CT-based attenuation correction, scatter correction, and resolution recovery carried out on a Xeleris imaging workstation (version 4.0, GE Healthcare, Waukesha, WI, USA). The reconstructed images had a matrix size of 128 × 128 with a section thickness of 4.42 mm. The minimal source-to-collimator distance for the parallel-hole collimation of Tc-99m was set to 4 mm. The camera sensitivity of the scanner was calibrated as 68.06 count/second/Mbq, using a dedicated point source provided by GE healthcare.

The patient information and acquisition parameters were obtained at the time of injection. Patient height and body weight was measured prior to injection. The pre-injection and post-injection activity of the syringe was measured before and after injection, respectively. The time of each measurement was also recorded. The injected radioactivity was automatically calculated with a decay correction on the Xeleris workstation as follows:

Injected radioactivity = post-injection activity − pre-injection activity

### 2.3. Image Analysis

#### 2.3.1. SPECT Image Evaluation

All images were evaluated by experienced nuclear medicine physician blinded to histological results. All SPECT/CT images were evaluated on a dedicated workstation (Xeleris 4.0, GE Healthcare, Waukesha, WI, USA) that displayed CT, SPECT, and fused SPECT/CT images. For quantitative analysis, the volumes of interest (VOIs) were generated by automatic segmentation function on the dedicated workstation by clicking the seed point on the tumor center. The generated VOI was manually inspected and corrected if needed. Quantitative parameters were obtained from VOIs using the Q.Metrix toolkit installed on the dedicated workstation. SUVmax in a given VOI was calculated as follows:

SUVmax = (maximum radioactivity/voxel volume)/(injected radioactivity/bodyweight) (g/mL)

#### 2.3.2. CT Image Evaluation

**Figure 1:**
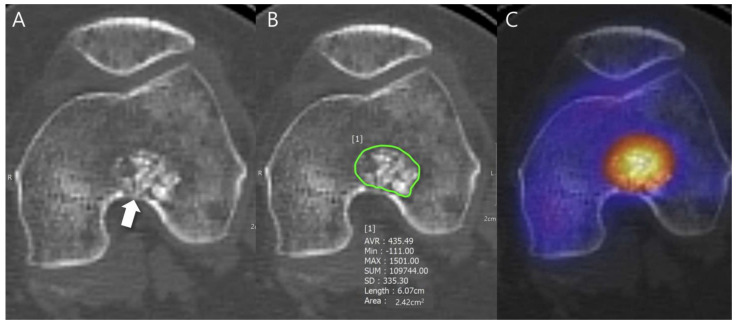
Radiodensity measurement in a patient with ACT in the distal femur. (**A**) Axial CT image shows a lobulated mass containing chondroid matrix mineralization and focal deep endosteal scalloping ≥ 2/3 of the normal cortical thickness (arrow). (**B**) Mean (±SD) attenuation of this lesion was measured using the freehand ROI tool (green line) and HU values were found to be 435.49 ± 335.30 [−111 to 1501]. (**C**) Fused SPECT/CT image shows the radioactive uptake with SUVmax of this lesion which was calculated to be 23.71.

First, radiodensity measurements were performed using a picture archiving and communication system (PACS) workstation (Zetta PACS, TaeYoung Soft, Anyang-si, Republic of Korea). The independent evaluation of images was performed by two musculoskeletal radiologists. Both readers were blinded regarding clinical information including surgery and histopathological results. A region of interest (ROI) marker was placed around the lesion with the use of the freehand ROI tool, which produced the maximum, minimum, mean, and standard deviation (SD) values of the lesions’ radiodensity in Hounsfield units (HU; HUmax, HUmin, HUmean, and HU_SD_). The CT slice on which the lesion had the largest cross-sectional area was selected. Among these, the axial slice with an abundant mineralized matrix and increased radionuclide uptake as seen on fused SPECT/CT images were selected. The freehand ROI was drawn to contain the lesion only within the intramedullary canal, ensuring that the bony cortex was not included within the ROI. When the lesion margin was not well visualized, the fused SPECT/CT image or MRI were referenced (Figure 1). 

**Figure 2:**
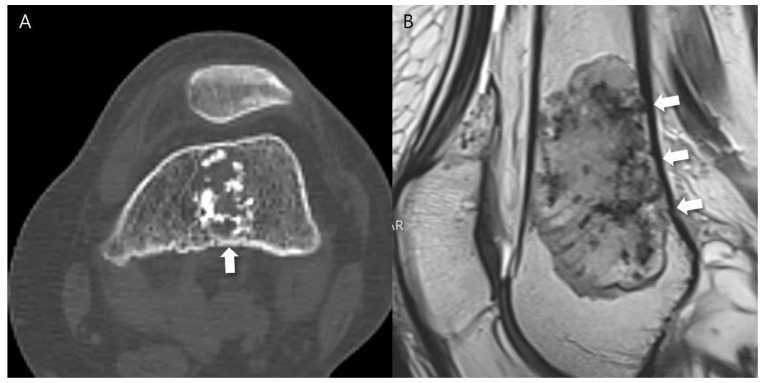
Extensive endosteal scalloping ≥ 2/3 of the lesion length in a patient with ACT in the distal femur. (**A**) An axial CT image shows a lobulated mass containing chondroid matrix mineralization and focal endosteal scalloping (arrow). (**B**) A sagittal T2-weighted image shows a lobulated mass with heterogeneously increased signal intensity and extensive endosteal scalloping ≥ 2/3 of the lesion length (arrows).

**Figure 3:**
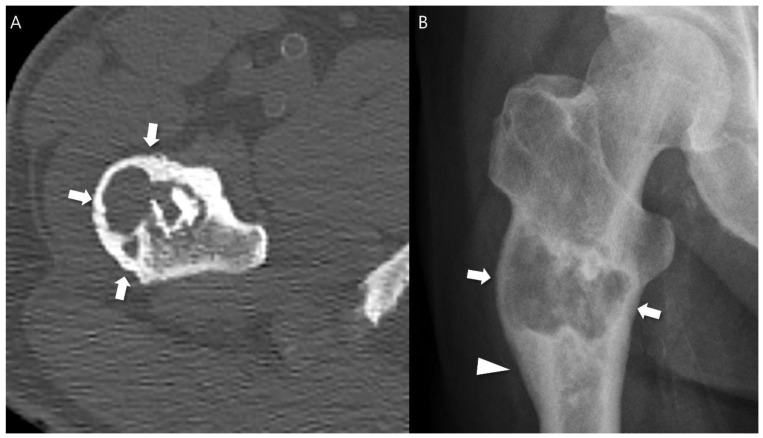
Expansile cortical remodeling in a patient with CS2 in the proximal femur. (**A**) An axial CT image shows a lobulated mass containing chondroid matrix mineralization and expansile cortical remodeling (arrows). (**B**) A plain radiograph shows a lobulated mass with expansile cortical remodeling (arrows) with cortical thickening (arrowhead).

**Figure 4:**
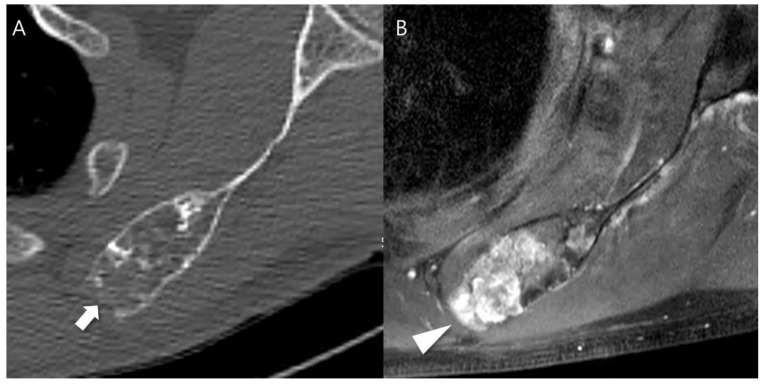
Cortical destruction with small extraosseous soft tissue extension in a patient with CS2 in the scapula. (**A**) An axial CT image shows a lobulated mass containing chondroid matrix mineralization and focal cortical destruction (arrow). (**B**) An axial T1-weighted fat-suppressed enhanced MRI shows a lobulated mass with extraosseous soft tissue extension (arrowhead).

Any aggressive CT features for the grading of central cartilaginous bone tumors including (i) deep endosteal scalloping ≥ 2/3 of the normal cortical thickness (Figure 1), (ii) extensive endosteal scalloping ≥ 2/3 of the lesion length (Figure 2), (iii) expansile cortical remodeling (Figure 3), and (iv) cortical destruction with or without soft tissue extension (Figure 4) were also evaluated. The CT images were evaluated in conjunction with the plain radiographs and/or MRI. After finishing the independent review, a consensus review of the CT was performed. The two radiologists reviewed the CT images together to reach a final consensus on discrepant interpretations from the independent reading.

### 2.4. Statistical Analyses

To reveal the correlative relationships between SUVmax and radiodensity measurements in HU, the Pearson’s correlation coefficient was calculated. Strength of correlation was interpreted as follows: Spearman’s rho (denoted as *r*)—0.0 to 0.1 no correlation, 0.1 to 0.3 poor correlation, 0.3 to 0.5 fair correlation, 0.5 to 0.7 moderate correlation, 0.7 to 1 very strong correlation, and 1 perfect correlation. Assessment of the relative importance of regressors in the multiple linear regression analysis was performed. A student’s *t*-test was performed to compare SUVmax and radiodensity measurements in HU, and a chi-square test was conducted to compare aggressive CT features of central cartilaginous bone tumors between the benign group (enchondromas) and the malignant group (low-to-intermediate grade chondrosarcomas). Intraclass correlation coefficients (ICCs) were used to evaluate interobserver reliability for the SUVmax and radiodensity measurements in HU. The ICC was calculated using a two-way random model by absolute agreement. The degree of agreement was interpreted as follows: ICC < 0.40 poor, 0.4–0.59 fair, 0.60–0.74 good, 0.75–1.00 excellent. Interobserver variability for the aggressive CT features of central cartilaginous bone tumors was assessed using kappa statistics. A kappa value lower than 0.40 indicated poor agreement, 0.40 to 0.59 moderate agreement, 0.60 to 0.79 good agreement, and 0.80 or greater excellent agreement. For all statistical comparisons, the significance level was set to *p* < 0.05. Statistical analysis was conducted using a software package (SPSS v. 20.0, Chicago, IL, USA). 

## 3. Results

### 3.1. Relationship of SUVmax with Radiodensity in HU Measurements

**Figure 5:**
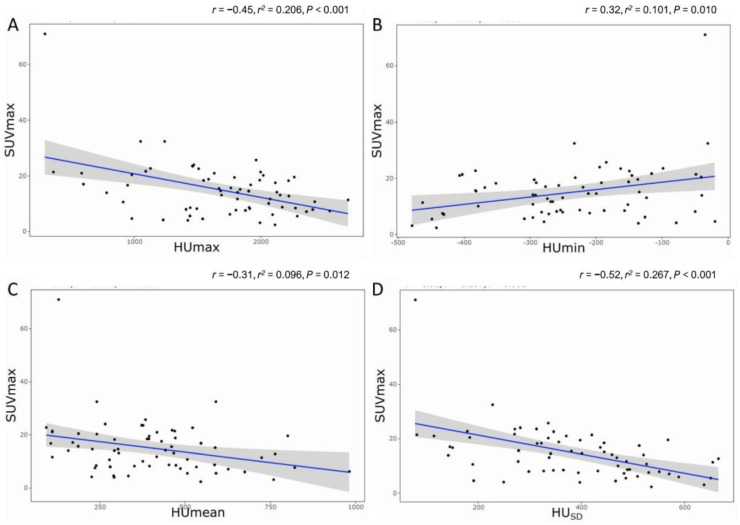
The relationship between SUVmax and radiodensity. (**A**) HUmax, (**B**) HUmin, (**C**) HUmean, and (**D**) HU_SD_. *r* = Pearson’s correlation; *r*^2^ = goodness-of-fit of linear regression.

Pearson’s correlation analysis showed that SUVmax demonstrated a fair negative correlation with HUmax (*r* = −0.45, *p* < 0.001), a fair positive correlation with HUmin (*r* = 0.32, *p* =0.010), a fair negative correlation with HUmean (*r* = −0.31, *p* = 0.012), and a moderate negative correlation with HU_SD_ (*r* = −0.52, *p* < 0.001). Figure 5 shows these relationships with the linear fit trend lines and *r*^2^ values (goodness-of-fit of linear regression). The multiple linear regression analysis demonstrated that the HU_SD_ (*r* = −0.52, *r*^2^ = 0.256, *p* < 0.001) was significantly and independently associated with SUVmax.

The interobserver reliability of the radiodensity between both readers were ‘excellent’ for HUmax (ICC of 0.861, *p* < 0.001), HUmean (ICC of 0.933, *p* < 0.001), and HU_SD_ (ICC of 0.944, *p* < 0.001), and ‘fair’ for HUmin (ICC of 0.710, *p* = 0.001).

### 3.2. Association of SUVmax and CT Features between Benign and Malignant Groups

Since most of the relationships between radiodensity in HU measurements and SUVmax showed a negative correlation, further subgroup analysis was performed to determine the association of each parameter between the benign (enchondroma) and malignant (ACT/CS1 + CS2) groups. 

First of all, most of the aggressive CT features of central cartilaginous bone tumors were significantly more frequent in the malignant group than in the benign group (deep endosteal scalloping, 60.0% vs. 11.1%, *p* < 0.001; extensive endosteal scalloping, 80.0% vs. 26.7%, *p* < 0.001; expansile cortical remodeling, 35.0% vs. 2.2%, *p* = 0.001) (Table 1). Interobserver agreement between the two readers was ‘good’ for deep endosteal scalloping (κ, 0.710) and extensive endosteal scalloping (κ, 0.757), and ‘excellent’ for expansile cortical remodeling (κ, 0.867) and cortical destruction (κ, 0.936).

**Figure 6:**
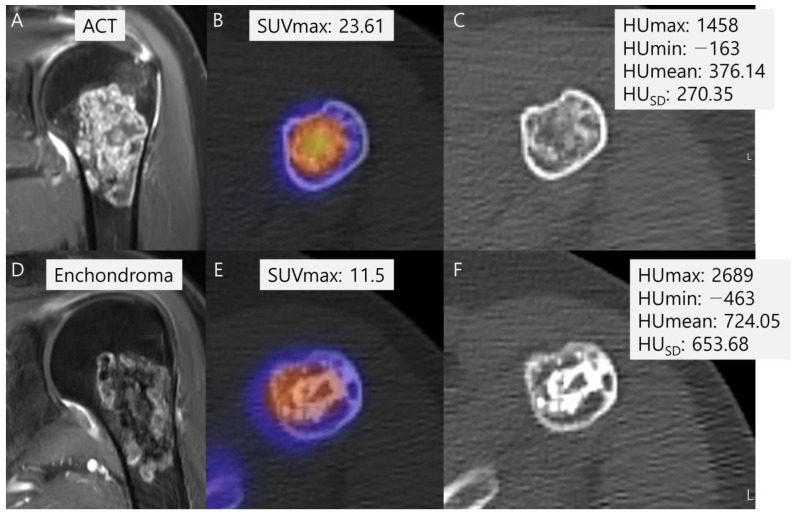
Comparison of SUVmax and radiodensity in HU measurements between ACT (**A**–**C**) and enchondroma (**D**–**F**). (**A**) A coronal T1-weighted fat-suppressed enhanced MRI shows a lobulated mass with septal enhancement with pathology of ACT. (**B**) A fused SPECT/CT image shows the radioactive uptake with SUVmax of this lesion which was calculated to be 23.61. (**C**) The radiodensity in HU measurement of this lesion was found to be 376.14 ± 270.35 [−163 to 1458]. (**D**) A coronal T1-weighted fat-suppressed enhanced MRI shows a lobulated mass with faint septal enhancement with pathology of enchondroma. (**E**) A fused SPECT/CT image shows the radioactive uptake with SUVmax of this lesion which was calculated to be 11.5. (**F**) The radiodensity in HU measurement of this lesion was found to be 724.05 ± 653.68 [−463 to 2689].

SUVmax was also significantly higher in the malignant group than in the benign group (22.3 ± 13.2 vs. 11.8 ± 5.9, *p* = 0.003) (Table 2). Among the radiodensity measurements in HU, only HU_SD_ was significantly lower in the malignant group than in the benign group (322.5 ± 149.1 vs. 405.1 ± 140.3, *p* = 0.036) (Table 2). In addition, HUmax (1522.6 ± 623.4 vs. 1748.8 ± 480.9, *p* = 0.116) and HUmean (381.5 ± 193.3 vs. 412.8 ± 197.9, *p* = 0.556) were lower in the malignant group than in the benign group, although without statistically significant differences, and HUmin (−223.0 ± 137.2 vs. −244.2 ± 113.1, *p* = 0.515) was higher in the malignant group than in the benign group, again without statistically significant difference (Table 2). A representative case is shown in Figure 6.

## 4. Discussion

Our findings revealed a significant negative correlation between SUVmax and radiodensity measurements in HU, including HUmax, HUmean, and HU_SD_. This contradicts the assumption that the strength and distribution of chondroid matrix mineralization directly influences SUVmax, suggesting an inverse relationship instead. To further explore this paradoxical association, subgroup analyses were conducted. The data were divided into benign (enchondroma) and malignant (low-to-intermediate grade chondrosarcoma) groups, and comparisons were made in relation to SUVmax and radiodensity measurements in HU between the two groups. The subgroup analysis showed significantly higher SUVmax in the low-to-intermediate grade malignant group, which exhibited more frequent CT aggressive features compared to the benign group. It also revealed significantly lower HU_SD_ in the low-to-intermediate grade malignant group compared to the benign group. Although statistical significance was not established, a trend of lower radiodensity of HUmax and HUmean was observed in the low-to-intermediate malignant group relative to the benign group. 

Bone scintigraphy uptake in cartilaginous tumors typically increase with a higher-grade [17]. This is thought to reflect the cortical destruction and bone permeation, as important histological features of high-grade tumors include infiltration and encasement of the existing trabecular bone [18]. Increased bone radiotracer uptake can be objectively described by SUV in SPECT/CT images. Previous research by Choi et al. [10] demonstrated that ACT exhibits a higher SUVmax compared to enchondroma. This suggests that the higher SUVmax in ACT may reflect a greater degree of reaction in the surrounding bone, such as cortical extension or permeation, as observed in ACT, leading to increased radionuclide uptake [17]. However, SUVmax can be influenced by several factors, including BMD which is known to be positively correlated with SUVs [19]. Therefore, there was curiosity regarding whether chondroid matrix mineralization might influence the SUVmax of tumors. This curiosity led us to initiate a study correlating radiodensity in HU measurements with SUVmax of central cartilaginous bone tumors. 

Based on existing literature, a positive correlation between SUVmax and the radiodensity of the tumor matrix was anticipated [19]; however, our actual results contradicted our expectations. Our findings revealed a significant negative correlation between SUVmax and radiodensity measurements in HUmax, HUmean, and HU_SD_. This result led us to the discovery that the strength and distribution of chondroid matrix mineralization do not directly influence SUVmax; instead, they exhibit an inverse relationship. Consequently, further subgroup analysis was prompted. 

Subgroup analysis was conducted, dividing the patients into two groups: benign (enchondroma) and low-to-intermediate grade malignant (ACT/CS1 + CS2) groups. SUVmax and radiodensity measurements in HU were subsequently compared between the two groups. The subgroup analysis revealed significantly higher SUVmax in the malignant group, which exhibited more frequent CT aggressive features compared to the benign group. This finding is consistent with previous studies aiming to differentiate benign from malignant cartilaginous bone tumors using bone SPECT/CT [10,20]. In bone SPECT/CT, SUVmax serves as an indicator of osteoblastic activity, reflecting cortical destruction and permeation in cartilaginous bone tumors. Our results confirm that low-to-intermediate grade chondrosarcomas are associated with higher SUVmax and more frequent CT aggressive features, such as deep and extensive endosteal scalloping and expansile cortical remodeling [21,22,23]. 

Subgroup analysis also revealed a significantly lower HU_SD_ in the low-to-intermediate grade malignant group compared to the benign group. Although statistical significance was not established, a trend of lower radiodensity was observed in the low-to-intermediate grade malignant group (HUmax and HUmean). Balta et al. conducted a study analyzing cartilaginous bone tumors using HU measurements and reported that ACT exhibited lower HU values than enchondroma. However, their study analyzed only the maximum and minimum values of HU without the SD of HU [15]. Although our study showed statistical significance only in HU_SD_, and not in HUmax, HUmin, and HUmean, between the two groups, there appears to be some degree of similarity in the results between the study by Balta and ours. In other words, a higher SD of HU in a tumor matrix with dense calcification indicates a greater likelihood of a stable enchondroma, while a lower SD of HU in a less calcified tumor matrix suggests a greater likelihood of an active aggressive low-to-intermediate grade chondrosarcoma [15]. 

Relevant previous studies have demonstrated that HU measurements in CT examinations, including the proximal femur and lumbar vertebrae, can predict BMD and strength [24,25] as well as bone neoplasms [26,27,28,29]. Thus, it can be concluded that HU measurements on CT scans can serve as a tool to differentiate between enchondroma and ACT, reflecting the characteristics of the tumor matrix. This aspect will be further analyzed in the future with a larger sample size and additional tools such as texture analysis [30,31,32,33]. It is also notable that, in the combined modality of SPECT/CT, both radiotracer and radiodensity information can be obtained simultaneously without additional examinations. Performing multiple examinations during disease evaluation increases the medical cost and decreases patient satisfaction due to the need for repeated visits to the hospital. By reducing the total number of examinations, SPECT/CT may improve patient convenience and reduce the total medical cost.

There are several limitations in this study. Firstly, it is retrospective in nature and conducted at a single center. Larger studies are required to confirm the results in the future. Secondly, there is a lack of histological confirmation for some portions of the enchondromas. Instead, we consider the diagnosis of enchondroma based on radiological imaging and follow-up results to be clinically valid. Third, the study did not encompass grade 3 chondrosarcoma (high-grade malignancy), thus limiting the capacity to definitely establish a conclusive relationship among chondrosarcoma malignant features, SUVmax, and HU values. Therefore, our results derive from the incorporation of low-to-intermediate grade chondrosarcomas only, with plans to broaden the analysis by including more patients across multiple centers in the future. Lastly, the heterogeneous tumor locations, such as in the trunk, pose a limitation. Future studies will include a large number of patients with tumors located only in the extremities or only in the trunk. 

## 5. Conclusions

The current study revealed a negative correlation between SUVmax and radiodensity in HU measurements of the tumor matrix in central cartilaginous bone tumors. It was observed that higher SUVmax and lower HU_SD_ were associated with a higher probability of having a malignant cartilaginous bone tumor (low-to-intermediate grade) with aggressive feature and a less calcified tumor matrix. This study highlights the potential usefulness of SPECT/CT scans to tumor diagnosis and characterization for central cartilaginous bone tumors. Further research is needed to validate these findings in larger patient cohorts and to explore their clinical applications. 

## Figures and Tables

**Table 1:** Comparison of aggressive CT features between benign and malignant groups.

	Benign, Enchondroma	Malignant, ACT/CS1 + CS2	*p* Value
	(n = 45)	(n = 20)	
Deep endosteal scalloping			<0.001
<1/3 of normal cortical thickness	40 (88.9%)	8 (40.0%)	
≥2/3 of normal cortical thickness	5 (11.1%)	12 (60.0%)	
Extensive endosteal scalloping			<0.001
<1/3 of the lesion length	33 (73.3%)	4 (20.0%)	
≥2/3 of the lesion length	12 (26.7%)	16 (80.0%)	
Expansile cortical remodeling			0.001
Absent	44 (97.8%)	13 (65.0%)	
Present	1 (2.2%)	7 (35.0%)	
Cortical destruction			0.169
Absent	45 (100.0%)	18 (90.0%)	
Present	0 (0.0%)	2 (10.0%)	

**Table 2:** Comparison of SUVmax and radiodensity in HU measurements between benign and malignant groups.

	Benign, Enchondroma	Malignant, ACT/CS1 + CS2	*p* Value
	**(n = 45)**	**(n = 20)**	
SUVmax	11.8 ± 5.9	22.3 ± 13.2	0.003
HUmax	1748.8 ± 480.9	1522.6 ± 623.4	0.116
HUmin	−244.2 ± 113.1	−223.0 ± 137.2	0.515
HUmean	412.8 ± 197.9	381.5 ± 193.3	0.556
HU_SD_	405.1 ± 140.3	322.5 ± 149.1	0.036

## Data Availability

Dataset available on request from the authors.

## Abbreviations

ACT	Atypical cartilaginous tumor
BMD	Bone mineral density
CS1	Chondrosarcoma grade 1
CS2	Chondrosarcoma grade 2
CT	Computed tomography
HDP	Hydroxymethylene diphosphonate
HU	Hounsfield units
ICC	Intraclass correlation coefficients
MRI	Magnetic resonance imaging
SD	Standard deviation
SPECT	Single photon emission computed tomography
SUVmax	Maximum standardized uptake value
PACS	Picture archiving and communication system
ROI	Region of interest
VOI	Volume of interest
